# Application of CRISPR-Cas System to Mitigate Superbug Infections

**DOI:** 10.3390/microorganisms11102404

**Published:** 2023-09-26

**Authors:** Ali A. Rabaan, Mona A. Al Fares, Manar Almaghaslah, Tariq Alpakistany, Nawal A. Al Kaabi, Saleh A. Alshamrani, Ahmad A. Alshehri, Ibrahim Abdullah Almazni, Ahmed Saif, Abdulrahim R. Hakami, Faryal Khamis, Mubarak Alfaresi, Zainab Alsalem, Zainab A. Alsoliabi, Kawthar Amur Salim Al Amri, Amal K. Hassoueh, Ranjan K. Mohapatra, Kovy Arteaga-Livias, Mohammed Alissa

**Affiliations:** 1 Molecular Diagnostic Laboratory, Johns Hopkins Aramco Healthcare, Dhahran 31311, Saudi Arabia; 2 College of Medicine, Alfaisal University, Riyadh 11533, Saudi Arabia; 3 Department of Public Health and Nutrition, The University of Haripur, Haripur 22610, Pakistan; 4 Department of Internal Medicine, King Abdulaziz University Hospital, Jeddah 21589, Saudi Arabia; 5 Infectious Disease Division, Department of Internal Medicine, Dammam Medical Complex, Dammam 32245, Saudi Arabia; 6 Bacteriology Department, Public Health Laboratory, Taif 26521, Saudi Arabia; 7 College of Medicine and Health Science, Khalifa University, Abu Dhabi 127788, United Arab Emirates; 8 Sheikh Khalifa Medical City, Abu Dhabi Health Services Company (SEHA), Abu Dhabi 51900, United Arab Emirates; 9 Department of Clinical Laboratory Sciences, College of Applied Medical Sciences, Najran University, Najran 61441, Saudi Arabia; 10 Department of Clinical Laboratory Sciences, College of Applied Medical Sciences, King Khalid University, Abha 62223, Saudi Arabia; 11 Infection Diseases Unit, Department of Internal Medicine, Royal Hospital, Muscat 1331, Oman; 12 Department of Pathology and Laboratory Medicine, Zayed Military Hospital, Abu Dhabi 3740, United Arab Emirates; 13 Department of Pathology, College of Medicine, Mohammed Bin Rashid University of Medicine and Health Sciences, Dubai 505055, United Arab Emirates; 14 Department of Epidemic Diseases Research, Institute for Research and Medical Consultations (IRMC), Imam Abdulrahman Bin Faisal University, Dammam 31441, Saudi Arabia; 15 Pharmacy Department, Qatif Central Hospital, Qatif 32654, Saudi Arabia; 16 Infection and Control Department, Armed Forces Hospital, Azaibah 130, Oman; 17 Pharmacy Department, King Saud Medical City, Riyadh 7790, Saudi Arabia; 18 Department of Chemistry, Government College of Engineering, Keonjhar 758002, India; 19 Escuela de Medicina-Filial Ica, Universidad Privada San Juan Bautista, Ica 11000, Peru; 20 Escuela de Medicina, Universidad Nacional Hermilio Valdizán, Huanuco 10000, Peru; 21 Department of Medical Laboratory Sciences, College of Applied Medical Sciences, Prince Sattam Bin Abdulaziz University, Al-Kharj 11942, Saudi Arabia

**Keywords:** superbugs, CRISPR/Cas, base editing, CBE, ABE, base editors

## Abstract

Multidrug resistance in bacterial strains known as superbugs is estimated to cause fatal infections worldwide. Migration and urbanization have resulted in overcrowding and inadequate sanitation, contributing to a high risk of superbug infections within and between different communities. The CRISPR-Cas system, mainly type II, has been projected as a robust tool to precisely edit drug-resistant bacterial genomes to combat antibiotic-resistant bacterial strains effectively. To entirely opt for its potential, advanced development in the CRISPR-Cas system is needed to reduce toxicity and promote efficacy in gene-editing applications. This might involve base-editing techniques used to produce point mutations. These methods employ designed Cas9 variations, such as the adenine base editor (ABE) and the cytidine base editor (CBE), to directly edit single base pairs without causing DSBs. The CBE and ABE could change a target base pair into a different one (for example, G-C to A-T or C-G to A-T). In this review, we addressed the limitations of the CRISPR/Cas system and explored strategies for circumventing these limitations by applying diverse base-editing techniques. Furthermore, we also discussed recent research showcasing the ability of base editors to eliminate drug-resistant microbes.

## 1. Introduction

Since the discovery of the first antibiotics, researchers have prioritized the development of novel strategies to use against bacterial infections. The world has certainly been forced to implement considerable obstacles to tackle this issue because the propensity of disease-causing microbes to acquire antibiotic resistance has swiftly expanded. Ergo, superbugs are addressed in light of this; these are bacterial strains that have evolved resistance to a variety of medications used to treat common ailments. According to their ability to resist infection, the World Health Organization (WHO) classified antibiotic-resistant bacteria into 12 separate groups in 2017. Among this group, *A. baumannii*, *P. aeruginosa*, and *Enterobacter* spp. were included on the list of the most critical pathogens that were found to be responsible for causing high mortality in hospital patients [1]. The improper use and overuse of antibiotics are two factors that contributed to the rise of superbugs [2]. Other factors also included the ways that bacteria gain resistance to antimicrobial drugs, some of which are highlighted here, such as random gene mutation [3], the transmission of resistance genes via horizontal gene transfer (HGT) [4], altering the drug permeability or enhancing the drug efflux [5], and having the potential to form biofilms [6]. Bacterial populations that cause long-term infections have the potential to become tolerant to antibiotics due to the presence of persister cells, which can result in chronic infections without any change to the bacteria’s genetic makeup. Because of the multifaceted nature of their transmission, there is an urgent need to develop innovative approaches for detecting, treating, and combating superbug infections.

Several scientific studies have emphasized the harmful nature of superbugs and a variety of ways to deal with them [7,8,9].

To date, various therapeutic approaches have been used to target the multidrug resistance mechanisms exhibited by superbugs, including the development of next-generation antibiotics, phage therapy as a promising alternative to antibiotics, the production of host defense peptides (an alternative to conventional antibiotics), the use of plant-derived products like essential oils and medicinal extracts, and the use of quorum sensing inhibitors (QSIs) and efflux pump inhibitors (EPIs) [10,11,12]. However, each of these traditional methods has drawbacks [13], and at the moment the FDA has passed judgment against the use of phage-based therapy due to safety concerns [14].

Thus, to mitigate the emergence of superbugs’ resistance with minimal negative impacts on humans or on the environment, CRISPR/Cas-based approaches have become a widely adopted, hopeful alternative to conventional approaches. In comparison with other DNA-based gene engineering techniques, CRISPR-Cas genome editing has been hailed as being simple, adaptable, effective, and lacking the need for any specific markers to identify the species of pathogenic bacteria. Despite this, it has the ability to precisely target a specific sequence with a single guide RNA (gRNA) and its associated protein (Cas). Typically, this system serves as an adaptive immunity, providing protection against foreign genetic material [15]. For superbugs, on the other hand, it has a different mechanism of action, acting as a transferable and integrative system to target antibiotic-resistant genes. It induces DSBs (double-strand breaks) within the DNA of resistant bacteria and transforms them into antibiotic-sensitive ones [16].

The invention of CRISPR-based genome editing has had a significant impact on the medical field since it allows for the effective and precise identification and modification of the genes responsible for bacterial drug resistance. Previous studies demonstrated that by using the type II CRISPR-Cas9 system, a novel gene which makes bacteria highly resistant to the last-resort class of antibiotics was discovered and knocked out from *Escherichia coli* [17]. In *Mycobacterium tuberculosis*, a CRISPR-Cas-III-A system was used to target interference and crRNA processing using six *csm* and cas6 genes, respectively [18]. In essence, the CRISPR-Cas-III-A system was developed to build a powerful defense by identifying and targeting RNA and cleaving co-transcriptionally active DNA [19]. Additionally, genetic editing has been confirmed in a variety of antibiotic-resistant bacteria utilizing various CRISPR techniques, such as CRISPR-Cas-II-A for *Streptococcus agalactiae* [20], or a CRISPR-Cas12a strategy combined with nanozymes that is used to identify kanamycin-, ampicillin-, and chloramphenicol-resistant genes [21]. Despite its widespread use, conventional CRISPR-Cas-mediated gene editing has some drawbacks, including the creation of genomic instability due to inaccurate off- and on-target editing, which may be triggered by the low GC content of sgRNA, the use of protospacer adjacent motif (PAM-in) orientation, or using an ineffective delivery technique to eliminate AMR genes [22,23].

To address this issue, base-editing procedures are now being used to reduce the occurrence of the aforementioned errors. We can use this strategy to make many changes to DNA sequences without causing dsDNA cleavage. The two major components of base editors, which enable this technology to change bases accurately, are a Cas enzyme and an ssDNA for programmed DNA binding and a modifying enzyme for exact nucleotide modification, respectively. To improve site-directed mutagenesis efficiency, DNA base editors have fusion proteins (nickase Cas9, dead Cas9, or dead Cas12a/b) coupled to ssDNA-specific nucleobase deaminases [24]. This article discusses the CRISPR/Cas system’s procedures and limitations with a focus on base-editing techniques for battling superbugs.

It is important to learn that the CRISPR-Cas approach was preceded by several other gene-editing methods such as TALEN and ZFN. Zinc finger nucleases (ZFNs) and transcription activator-like effector nucleases (TALENs) have distinct drawbacks when compared with the CRISPR-Cas system. ZFNs and TALENs require the design and engineering of custom proteins for each specific DNA target. This process can be laborious, time-consuming, and costly, making them less accessible to many researchers. In contrast, CRISPR-Cas relies on RNA guides, which are easier and more cost-effective to design and synthesize. While all gene-editing methods can have off-target effects, ZFNs and TALENs are more prone to off-target cleavage due to the intricacies of protein–DNA binding. CRISPR-Cas systems, while not immune to off-target effects, have seen significant advancements in reducing off-target cleavage through improved guide RNA design and specificity-enhancing modifications. Furthermore, CRISPR-Cas is highly versatile and can be easily retargeted by simply changing the guide RNA sequence, allowing for efficient multiplexing and simultaneous editing of multiple genes. ZFNs and TALENs lack the same level of flexibility and may require significant reengineering for each new target. These features make CRISPR-Cas a relatively more reliable, resilient, and cost-effective method of gene editing.

CRISPR-Cas was first discovered in bacteria and archaea, where it was discovered to be involved in adaptive immunity [25]. This system can be used by bacteria and archaea to recognize and neutralize plasmids and viruses that are invading their cells. Over the past ten years, CRISPR-Cas has been modified for use in genetic engineering and gene editing. Base editing and CRISPR-Cas gene editing are two important techniques for accurately altering an organism’s DNA. The technology to treat genetic abnormalities [26], boost crop yields [27], and develop innovative biotech applications [28] is now available because of these advancements in technology. Because of technical advancements, scientists can now carry out research and experiments more effectively, which means results can be produced more quickly. Two recent developments in more precise technology are base editing and CRISPR gene editing.

## 2. CRISPR-CAS Gene Editing: A Brief Overview about Target Superbugs

CRISPR-Cas systems can be used to disable genes responsible for antibiotic resistance in superbugs, making them more susceptible to existing antibiotics and therefore easier to treat [29]. Additionally, they are used to introduce desirable traits in bacteria, such as the ability to produce antimicrobial compounds, allowing scientists to create “designer” bacteria that can be used to fight off superbugs. Until this point, several research studies have been published that altered antibiotic-resistant microorganisms using various CRISPR-Cas systems [16,30,31]. The type II CRISPR system is more efficient than the type I system because it is faster, more specific, and has a higher success rate. Additionally, this system can target multiple genes simultaneously, making it more versatile and effective. Each sub-type II system also consists of different orthologs, further broadening the range of applications for this technology. Orthologs are proteins that have similar structures and functions but are produced by different species. The details of each ortholog that are used for bacteria are given in Table 1.

**Figure 1:**
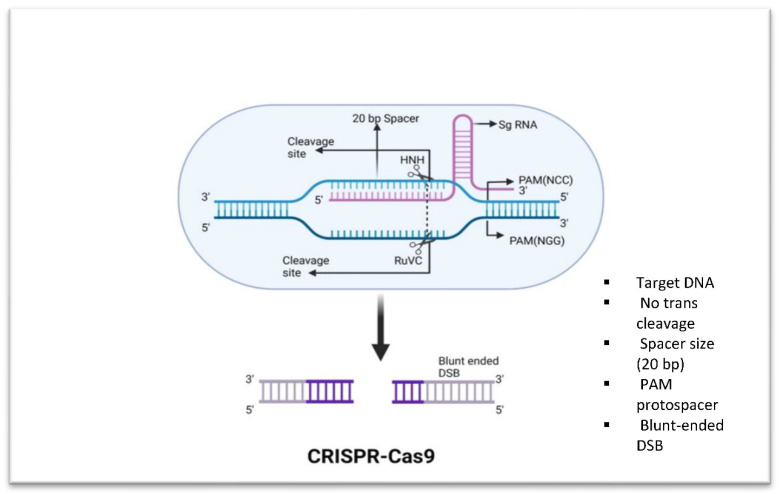
Fundamental components of CRISPR-Cas9. Cas9 is able to cleave dsDNA. Cas9 binds to a genomic region with a PAM sequence of 5′-NGG-3′ when guided by a sgRNA. This results in a double-stranded break.

The CRISPR/Cas9 multiplexed detection system (Figure 1) was developed by the Research Centre for Environmental and Management Sciences (RCEMS). Cas9-based DNA probes are used in this method to isolate AMR genes associated with gram-negative bacteria [23]. Antibiotic-resistant genes can be quickly and precisely identified, edited, and modified with the help of probes. Staphylococcus aureus is a common human pathogen, and Bikard et al. [32] found that Cas9 may alter virulence factors in this bacteria species to decrease it and protect animals from infection. CRISPR-Cas9 has been found to remove target genes from bacterial chromosomes in a variety of bacteria, including *Bacillus cereus* [33], Escherichia coli [34], Pseudomonas putida [35], and Streptococcus pneumonia [36]. Additionally, a fluorescent tag is used to indicate the presence of the target sequence, making it considerably easier to detect and count bacteria and their associated AMR genes. E. faecium strain E745 was used by Maat et al. [37] to investigate how removing the lacL gene impacted galactosidase activity by employing GFP as a fluorescent protein reporter. For more accurate and productive gene targeting, researchers are improving the CRISPR/Cas9 system. Nuclear localization signals have been added to either one or both of the protein ends that make up the system to help fight the issue of drug resistance in bacteria.

**Figure 2:**
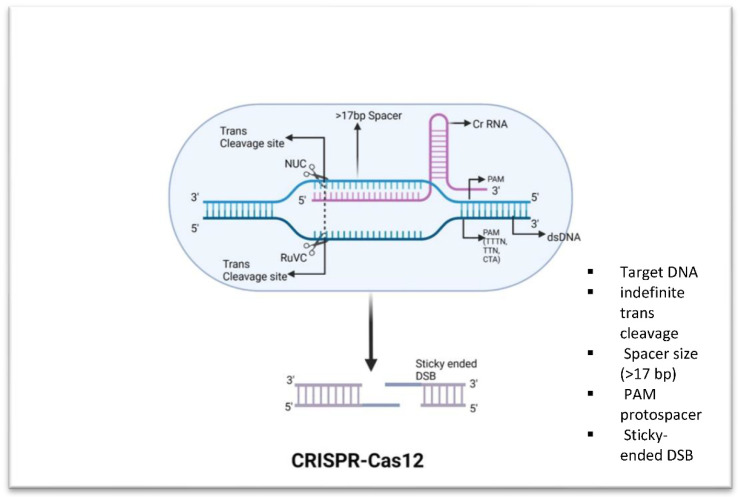
Fundamental components of CRISPR-Cas12. Cas12 has trans-collateral activity that allows it to cleave dsDNA that is not specific. Distal 5′-T-rich PAMs are recognized by Cas12 enzymes, which produce staggered double-strand breaks.

CRISPR/Cas12: This method is used to modify microbes in order to produce antibiotics and detect superbug populations, respectively. The technique involves using CRISPR-Cas12 to identify the DNA of the superbug, then using Cas12 to precisely target and destroy its genetic material. The Cas12 protein is a single-RNA guided endonuclease that processes its own guide RNAs to recognize and cut its target DNA. Cas12 does not need two RNAs like Cas9 does, nor does it possess inherent RNAse activity; instead, it does not require a tracrRNA for processing of the pre-crRNA into mature crRNA [38] (Figure 2). Cas12 also has a greater affinity for its target than Cas9, allowing for more precise and efficient targeting of genomic sequences. This makes it a popular choice for genome-editing applications [39]. A study by Li et al. [40] demonstrated the utility of a one-tube RPA-CRISPR/Cas12a platform for rapid detection of Methicillin-resistant *Staphylococcus aureus* (MRSA). Wang et al. [41] developed a platform with which they used multiplex amplification polymerase chain reaction together with the CRISPR/Cas12a approach in order to identify multidrug-resistant *Acinetobacter baumannii* (MDR-AB). Moreover, numerous studies have been documented that create CRISPR/Cas12a-based systems to combat different superbugs, including *Corynebacterium glutamicum* [42], *Klebsiella pneumonia* [43], and *Pseudomonas aeruginosa* [44].

**Figure 3:**
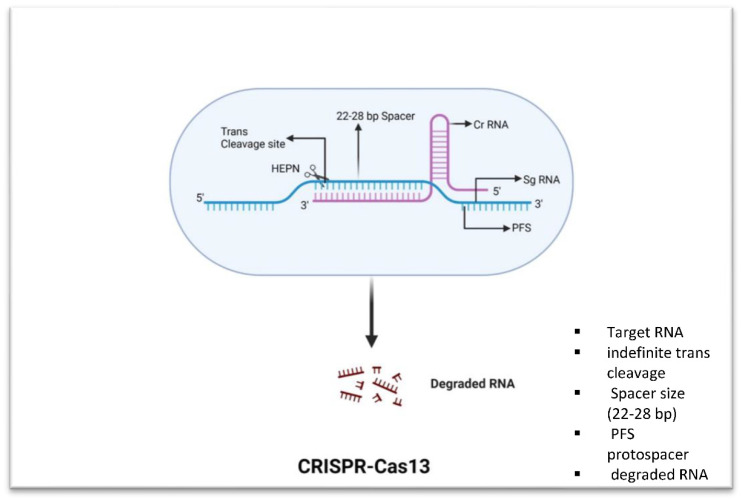
Fundamental components of CRISPR-Cas13. Cas13 has trans-collateral activity that allows it to cleave ssRNA that is not specific. It unleashes a nonspecific RNase activity after being activated by an ssRNA sequence with complementarity to its crRNA spacer breaks.

CRISPR/Cas13, on the other hand, can be used against both DNA and RNA viruses. It is possible to employ Cas13 to specifically target viral RNA and cleave it, preventing viral transcription and replication (Figure 3). Additionally, the system could be used to target specific bacterial genes or pathways, allowing for the development of targeted therapeutics that could be more effective than traditional antibiotics [45]. The results from a study by Kiga et al. [46] demonstrated that CRISPR-Cas13a was more effective than CRISPR-Cas9 at reducing the expression of IMP-1 (Metallo-beta-lactamase-1), a carbapenem-resistant gene that offers resistance to a broad range of beta-lactam antibiotics, including carbapenems. When used in combination with a crRNA targeting blaIMP-1, Cas13a was shown to lead to a 2–3-log decrease in bacterial cells. Their results suggest that CRISPR-Cas13a could be a viable alternative to CRISPR-Cas9 for treating bacterial infections caused by multidrug-resistant bacteria.

### Limitations of Type II CRISPR/CAS System for Precision Superbugs

While the CRISPR/Cas system has made significant strides forward, it still has several limits and restrictions that necessitate additional approaches for its resolution. Many different strategies have been proposed and used in an effort to solve technological problems. Because of their importance, especially in the context of treating superbugs, the technical issues in CRISPR/Cas-II technology are discussed in this section, along with the most recent attempts to address them.

Practically speaking, the CRISPR/CAS-II system is expensive and time-consuming to use in practice. This can be a major obstacle for researchers who are attempting to use the system to create precision superbugs. Due to collateral damage in the type II CRISPR/CAS system, it has limited specificity, meaning that off-target effects are more likely to occur. This can cause the superbug to undergo unexpected alterations, which might result in the emergence of new bacterial strains with higher antibiotic resistance [47]. Cas9-interrupted and NHEJ-repaired genes can lose their function. This can happen if the repair is incomplete, or if the repair results in a frame shift mutation that causes the gene to produce a nonfunctional protein [48]. Some limitations, like low survival rates of B. subtilis phages after Cas9 attack, were observed when using vector pRH030 [49]. It is possible that the large size of hp1 may have caused Cas9-induced indels to affect not only hp1, but also other nearby genes, disrupting cellular processes such as cell cycle, DNA replication, and DNA repair [50]. ScCas9, a Cas9 ortholog from *Streptococcus canis*, has recently been identified with a 5′NNG3′ PAM specificity, similar sequence homology to SpCas9 (89.2%), and comparable editing performance in both bacterial and human cells [51]. Furthermore, there are several fundamental limits of CRISPR-Cas12 and Cas13 diagnostics that must be considered when using these technologies for diagnostics. First, CRISPR-Cas12 and Cas13 enzymes require a relatively long target sequence, which can limit their specificity. For example, Cas12 enzymes typically require 20–30 base pairs in a sequence for recognition, while Cas13 enzymes typically require around 10 base pairs. This means that, in general, CRISPR-Cas12 and Cas13 diagnostics are more likely to detect similar but nontarget sequences, leading to false positive results [52]. Therefore, the aforementioned issues can be avoided using fundamental editing methods. In the fields of medicine, therapy, agriculture, and the natural sciences, base editing has already demonstrated enormous promise [53,54,55].

## 3. CRISPR/CAS Base Editing: A Brief Overview in Reference to Target Superbugs

Base editing is a technique that modifies the genome of living organisms without removing double-stranded DNA breaks, even though it makes precise changes to single nucleotide bases. The Cas9 endonuclease, in its modified form, is employed to enable this type of single-base editing of a nucleotide in a target DNA sequence. dCas9 protein is the term given to this modified Cas9 endonuclease [56]. dCas9 protein lacks nuclease activity, which implies it lacks the capacity to cut DNA, but it can still detect and attach to DNA when guided by guide RNA by employing a catalytically inactive Cas9 protein [57]. Base editors have the ability to precisely and effectively alter a single base in the bacterial genome, such as changing an A to a G, which would result in a mutation that would diminish the bacteria’s resistance to an antibiotic.

Biotinylated dCas9 can be used to study DNA–DNA interactions, chromatin control, epigenetic changes, and gene expression at a specific locus. These discoveries can be applied to research long-range DNA interactions, histone changes, and how chromatin affects gene expression [58]. To improve the effectiveness of gene expression, dCas9 can be fused with individual transcriptional activator domains (VP64, P65, and Rta) or a group (VPR) [59,60,61,62].

Additionally, dCas9 can be fused with gene suppressors such as KRABdCas9 in order to suppress gene expression [63,64]. Cas9 or Cas12a proteins can be fused with deaminase machinery to create powerful gene-editing tools. These enzymes allow editing at a single base pair level without the need for homology-directed repair (HR). Amidst being frequently utilized in animal cells, BEs have also been found in bacteria and plants, which has expanded the range of genome-editing applications. There are various constructs and mechanisms employed by developed BEs. BEs are used to facilitate transition mutations, converting C to T and A to G. CBEs specifically convert C to T, while ABEs specifically convert A to G (Table 2).

## 4. Classic to Latest Developments in DNA Base Editors

### 4.1. Cytosine Base Editors (CBEs) in Superbugs

**Figure 4:**
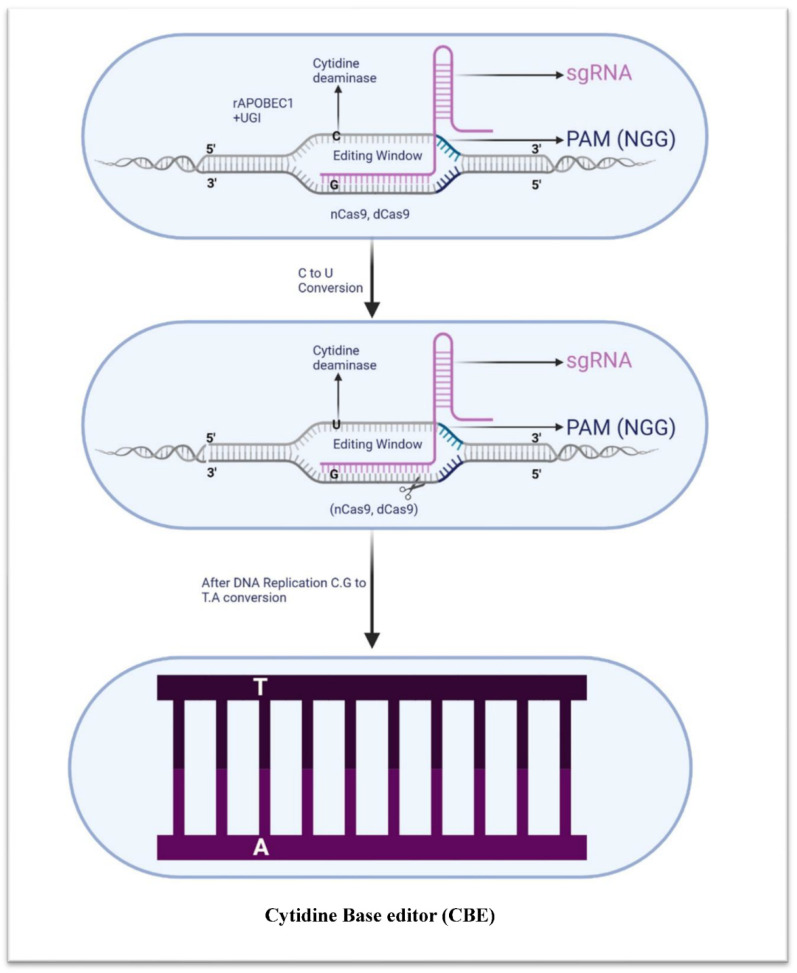
Schematic illustration of cytidine base editor. Cytidine base editor comprises cytidine deaminase (rAPOBEC1), uracil glycosylase inhibitor (UGI), and nickase Cas9 (nCas9, also known as dead Cas9). CBE is capable of inducing specific nucleotide changes, such as the transition of guanine to adenine or cytosine to thymine.

In 2016, the first report of a cytosine base editor was published, when a group of researchers at Harvard and MIT adapted the system of CRISPR-Cas9 to edit the single cytosine base in DNA [86]. The first generation of cytosine base editors (CBEs) utilizes Cas9 or dCas9 (catalytically inactive Cas9) to target a specific DNA sequence. The complex alters a cytosine in the sequence by changing it to a uracil, which is then interpreted by the replicative machinery as a thymine, thus achieving the desired mutation (Figure 4). The APOBEC family of enzymes is a group of naturally occurring cytidine deaminases found in vertebrates which can modify single-stranded DNA/RNA in order to protect the organism from viral invasion [87]. Previously, this first-generation CBE1 was reported in a number of drug-resistant bacteria, namely *Klebsiella pneumoniae* [88,89], *Pseudomonas aeruginosa* [90], *Staphylococcus aureus* [91], *Acinetobacter baumannii* [92], and *Streptomycetes* [65,93]. Although these base editors are more efficient, they can also introduce off-target edits and cytosine-to-uracil mutations at unwanted locations.

To overcome this limitation, alternative DNA-editing systems such as base editors 2 (BE2) and 3 (BE3) have been developed. These systems employ engineered Cas9 nickase (Cas9n) to target specific DNA sites, followed by the addition of a uracil-DNA glycosylase inhibitor, which prevents the UDG from cleaving the uracil and thus allows base editing to occur more effectively. Additionally, BE3 uses an engineered cytidine deaminase that is fused to Cas9n, allowing for more efficient and specific single-base editing [86]. A study examined the effects of a two-plasmid system on *Mycobacterium tuberculosis* (Mtb) [94]. The nickase Cas9-cytidine deaminase fusion protein approaches were further investigated for their effectiveness in modifying target sites in *Escherichia coli* and *Brucella melitensis*. This approach was employed to induce targeted mutagenesis in a number of genes in *E. coli*, including those involved in antibiotic resistance and metabolic pathways. In *B. melitensis*, the Cas9-cytidine deaminase fusion protein was utilized to bring about targeted mutagenesis in the gene responsible for the stimulation of virulence factors, resulting in the attenuation of the pathogen [66]. The pnCasSA–BEC system developed for *S. aureus* is useful for drug–target exploration; it is an accurate system for identifying potential drug candidates against this pathogen [91]. To increase the targeting scope of the editors, Zhang et al. [95] developed a modified version of St1Cas9 dubbed PAM-expanded St1Cas9 in order to widen the range of targets that editors can edit. A genome-editing technology using dCas9-CDA-ULstr was developed by Zhao et al. [79] to carry out multiplex genome editing in Streptomyces efficiently. This technology does not depend on homologous recombination (HR)-mediated DNA repair, thereby enabling effective functional genome research and metabolic engineering.

Furthermore, different Cas9 variants have been used in combination with BE3 to yield 2.5-fold higher editing efficiency. The *Staphylococcus aureus* Cas9 nuclease has been demonstrated to be effective when used with noncanonical PAMs. YEE-BE2 and YEE-BE3 are two cytidine deaminase mutants that have been developed to improve DNA selectivity and minimize off-target editing. YEE-BE3, a SaCas9 mutant, is the most efficient at DNA editing in a two-nucleotide window width, compared with YEE-BE2 and other noncanonical PAMs [68,69,70].

Moving onwards, BE4 and SaBE4 are fourth-generation base editors that combine rAPOBEC1 and Cas9D10A, two UGI molecules and amino acid linkers of varying lengths, to increase base-editing efficiency. This novel technique was first proposed by Komor et al. [86]. Recently, BE4 has been utilized to alter the genes of antibiotic-resistant bacteria and to explore the impacts of these changes on antibiotic susceptibility [71,72,73,74,75].

### 4.2. The Role of Adenine Base Editors (ABEs) in Superbugs

**Figure 5:**
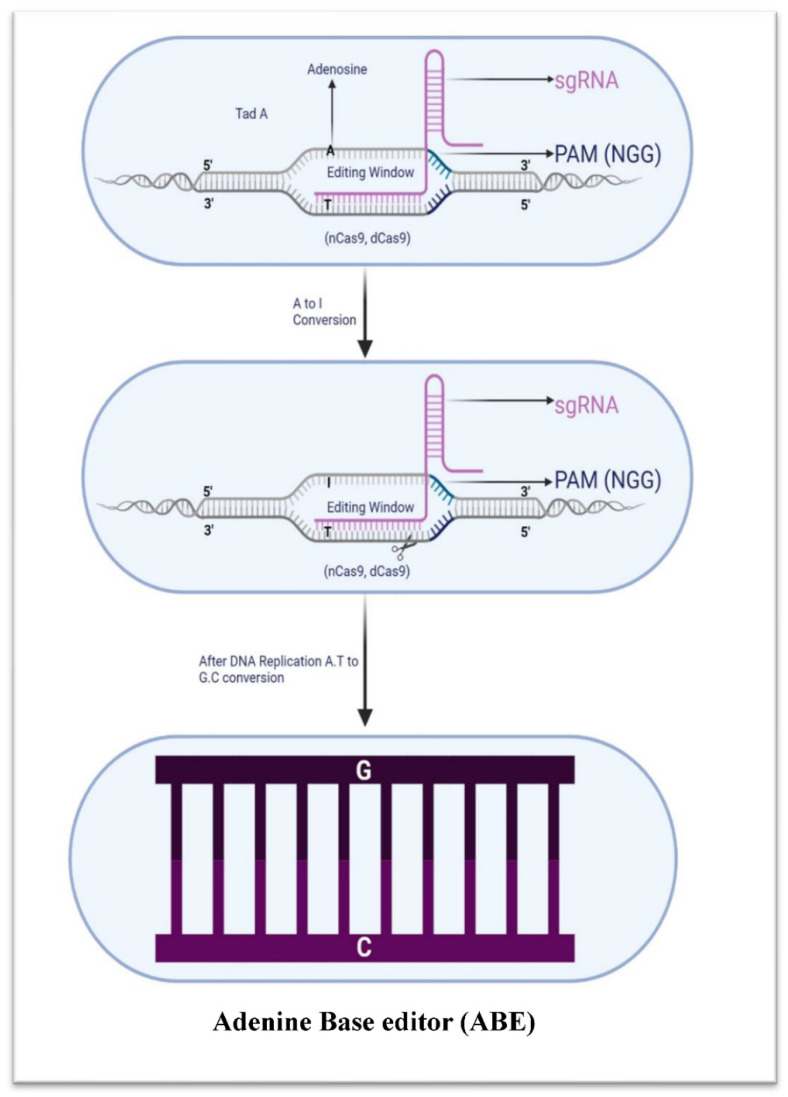
Adenine base editor schematic illustration (ABE). ABE uses an adenosine deaminase enzyme to convert A to I, by combining TadA and nCas9 or dCas9.

Gene-editing tools based on CRISPR technology, such as adenine base editors, allow for targeted alterations to a single nucleotide in the genome. Adenine deaminase and the Cas9 nuclease work together to change an A•T base pair into a G•C pair. This method can be utilized for the purpose of correcting and introducing mutations into the genome in a regulated manner [96] (Figure 5). The ability to make more precise and permanent changes to the genetic code is what makes adenine base editors more valuable than cytosine base editors. They are more efficient because they can make changes to multiple sites in a single reaction. Finally, adenine base editors are more flexible than cytosine base editors since they can edit both DNA and RNA. They can also be employed to alter or eliminate genes that code for virulence factors, thus decreasing a superbug’s infectious potential. The ABE developed by Liu et al. can identify and alter any base in RNA and DNA independent of the sequence. Aminoacylation and tRNA functions rely on the enzyme TadA, which in E. coli converts adenine to inosine in the anticodon loop of tRNA Arg. This is because adenine is the second most prevalent nucleotide in the codon–anticodon pairing, and forms a pair with guanine. As a result of TadA’s deamination of adenine to inosine, codon–anticodon pairing is improved, leading to more precise genetic code translation [96,97,98]. Several different parts of the ABE system work together to alter DNA base pairs. ABE-0 is a cytosine-to-uracil converting enzyme that uses Cas9–guide RNA and a cytidine deaminase as a single-component system. The two-component systems ABE-1 and ABE-2 use Cas9–guide RNA in conjunction with an adenine deaminase or uracil-DNA glycosylase to change adenines to inosines or uracil to thymine, respectively. The ABE-2 variants ABE-3 to ABE-7 each target a unique set of base pairs with a unique set of UDGs and gRNAs [96]. Bacterial and eukaryotic hosts are both considered to be viable environments for the ABE7.7, ABE7.8, ABE7.9, and ABE7.10 families of enzymes [83,84]. The active site changes in these ABEs increase their activity and increase their sequence compatibility, allowing them to cut a larger variety of DNA sequences than their predecessor.

Adenosine deaminase, which is involved in a form of gene-editing technique known as ABEs, converts adenosine (A) to inosine (I) at particular target DNA regions. Target sites 4 to 9 (ABE7.8 or ABE7.9) and 4 to 8 (ABE7.10) in human cells are successfully converted when this enzyme is fused to an inactive Cas9 mutant because of the ensuing transition from A•T to G•C at the target site [83], and this technique has been used to cleave plasmid DNA in a variety of bacteria, including gram-positive and gram-negative species, as well as antibiotic-resistant strains.

ABE8e is a next-generation adenine base editor that can be used with various Cas9 or Cas12 homologs to achieve superior editing results. Richter et al. [85] stated that ABE8e, an improved base editor, is much more active than ABE7.10 and offers a 590-fold increase in activity. It is also more processive, which could be useful for screening, disrupting regulatory regions, and multiplex base editing. On the other hand, ABE8e exhibits slightly greater levels of transcriptome-wide off-target RNA and DNA editing. A second mutation in the TadA-8e domain can fix this problem.

For the first time, the genome of a Pseudomonas species may be edited precisely and efficiently using a CRISPR-Cas-based base-editing method called dxABE-PS. It is made up of two parts: the deaminase-fused adenine base editor (ABE) and the xCas9 3.7 endonuclease. Since xCas9 3.7 can identify an NG PAM, it can be used to cut a wider variety of targets than previous versions of Cas9. The adenine base editor has been coupled with a deaminase so that it can alter A: T to G: C. The Pseudomonas genome may be edited precisely with an efficiency of up to one hundred percent using a combination of xCas9 3.7 and the adenine base editor [99].

Dual-base editors (ACBEs) were recently created to broaden the spectrum of DNA alterations. These ACBEs incorporate features from two separate base editors into a single tool, allowing C-to-T and A-to-G DNA substitutions to be made simultaneously [100,101,102]. The study by Shelake et al. [75] is important as it presents the first successful engineering of an improved activator-dependent CRISPR-Cas dual-base editor (iACBE) for microbial genome engineering. The PAM-relaxed nCas9-NG is used in iACBE4-NG to generate transversion and transition mutations at specific loci. This tool has been used to identify mutations in the rpoB gene that were not previously known and to confer resistance to rifampicin, demonstrating its usefulness for engineering microbial genomes and synthetic biology. Previously, desired features in *Bacillus subtilis* could be engineered utilizing programmable dual-deaminase base editors, such as CRISPR-ABE8e-CDA-nCas9. This platform could be exploited to enhance bacterial chassis for a variety of uses, including biofuel production, bioremediation, and drug development [103].

## 5. Delivery of Base Editors

The base-editing system may include modified guide RNAs, base editors, and delivery vehicles. The delivery vehicles must be designed to ensure that the components are efficiently and safely delivered into the nucleus of the target tissue. How base editors are delivered to cells is determined by both the type of base editor being utilized and the cell type that is targeted. Base editors can be introduced in vivo via injection or viral vectors like adeno-associated viruses (AAVs). Base editors can be administered ex vivo via electroporation, in which an electric field is used to form transient pores in the cell membrane, or via liposomes, which are small vesicles that carry the base editor into the cells.

### 5.1. Adeno-Associated Virus Vectors for In Vivo Delivery

AAV vectors can efficiently and precisely deliver base editors to target cells for gene editing. Customizing the vector allows researchers to modify the genomes of specific cell types without altering others, reducing off-target consequences [104]. More than 145 clinical trials using AAVs for gene therapy have been conducted so far, and three AAV-based medications (Glybera, Luxturna, and Zolgensma) have been authorized by the FDA. These advances clear the way for the development of novel therapeutics for a plethora of genetic illnesses [105,106]. Both CBEs and ABEs rely on adeno-associated viruses (AAVs), which are engineered to transport a specific gene or gene-editing tool, such as CRISPR-Cas9. It is common practice for scientists to create these recombinant AAVs in the lab by inserting a gene of interest into an AAV vector. The AAV6, AAV9, and AAV capsid serotypes 8, 9, rh10, and rh32 are widely employed for gene editing [107]. *Campylobacter jejuni* bacteria can be modified using small cjCBEmax and cjABE8e base editors injected by a single AAV [108]. Due to its nature as a viral vector, the AAV is limited in the amount of genetic material it can carry. Because of the size of the genes necessary for genome editing, delivering large genome editors like SpCas9 (4100 bp) or ABEs (5400 bp) is challenging. It becomes increasingly challenging to deliver the necessary genes to the target cell when the size of the genome editors becomes larger than the AAV capacity. This reduces the AAV’s utility as a gene delivery vector for extensive genome editing [109]. Thus, a dual-AAV system and the intein-mediated trans-splicing approach may help circumvent AAVs’ load restrictions. Intein-mediated trans-splicing utilizes a protein splicing element that can be inserted into a gene to join two separate sections of the gene together [110,111,112], while the dual-AAV system is an easy approach for delivering substantial portions of genetic material. These methods are successful against superbugs, and they may be utilized to create new and improved therapeutic agents for treating infections that have become resistant to existing drugs [113,114].

### 5.2. Nonviral Methods of Delivery

Base editors can also be delivered through nonviral delivery techniques like liposomes, nanoparticles, polymers, the electroporation approach, and CRISPR/Cas9 ribonucleoprotein complexes. Compared with viral vectors, these systems may be more efficient and less expensive [115]. The polymer-derivative CRISPR nanocomplex shows promise as a weapon against antibiotic-resistant bacteria. Using this technique, a CRISPR-Cas9 duo attached to a polymer-based nanoparticle is injected into a bacterial cell. The CRISPR-Cas9 complex can sever the target pathogen or antibiotic-resistant DNA sequence after it has been discovered. The study of this technique demonstrated that the Cr nanocomplex could easily penetrate the bacterial cell, connect to the bacterial membrane, and deliver the SpCas9 enzyme and sgRNA to the target region. The outcomes also showed that the Cr noncomplex successfully triggered genome editing in the bacterial genome, which resulted in a substantial decline in bacterial antibiotic resistance. This shows that the Cr noncomplex is an effective weapon in the struggle against bacteria that have evolved antibiotic resistance [116].

A popular method for introducing base editors into cells is electroporation. By temporarily increasing cell membrane permeability with electrical pulses, genetic material, such as base editors, can be delivered into the cell. When compared with other delivery techniques, this strategy has the advantages of speed, efficiency, and accessibility. Additionally, electroporation allows for the efficient transfer of large amounts of DNA, which increases editing effectiveness. Bacteria often withstand electroporation better than mammalian cells do because their cell walls are simpler and more rigid. Due to their material qualities, electric fields can pass through the single layer of peptidoglycan that makes up bacterial cell walls. This greatly expands the range of voltages and currents that can be used for electroporation without damaging the bacterial cell wall.

In contrast, mammalian cell membranes are quite thick due to the presence of many layers of phospholipids and proteins. To achieve effective electroporation, voltage and current must be precisely controlled [117]. Plasmids, vectors, and other genetic elements can be successfully introduced into bacteria through in vivo electroporation [118,119,120].

The use of carbon quantum dots (CQDs) as a nanomaterial for administering drugs is showing great promise. These materials are excellent for carrying pharmaceuticals and other bioactive chemicals because they are safe, biocompatible, and chemically stable. They have a high loading capacity and a large surface area, so they can carry more of the medications and bioagents that are needed to treat various conditions. Additionally, CQDs can prevent drug degradation and permit their continuous release [121,122,123,124,125,126,127,128,129].

## 6. Prospects of CRISPR Base Editing

CRISPR-Cas9, a technology discovered in 2012, has rapidly gained popularity as a means of DNA editing. Although this method has been praised as a possible answer to the most difficult superbug infections, it still has several limitations. The system’s limitations come from the fact that it primarily focuses on the root causes of superbugs, such as inadequate hygiene, crowding, and the use of drugs. It is unable to remove superbugs by aiming directly at them. Instead, the system kills germs by breaking their genetic material, which might render them harmless or significantly weaker. Since superbugs are so difficult to eradicate, the CRISPR-Cas technology can only be used to control their population size. In addition, the system cannot eliminate drug resistance in its entirety by focusing on individual genes responsible for the phenomenon. This means that the spread of antibiotic resistance will continue because it is impossible to target and destroy microorganisms that have developed resistance. Therefore, we propose considering these problems and obstacles from the following angles.

To maximize their effectiveness and minimize their off-target rate, researchers must first elucidate the precise mechanism of the CRISPR/Cas complexes, focusing on the recognition of target sequences through the use of Cas9/Cas12a and binding to DNA. Second, there needs to be more work put into the design of new CRISPR/Cas tools, especially those that make use of the endogenous system. Targeting specificity, efficiency, and accuracy are all factors that need to be taken into account while designing such tools. Third, more precise control approaches, such as the use of CRISPRi or CRISPRa to regulate gene expression, should be developed. These techniques allow for the precise regulation of gene expression, which has applications in the treatment of genetic illnesses and the modification of organisms. Finally, the CRISPR/Cas technology needs to be made safer for use. It is important to make sure the CRISPR/Cas system does not introduce any mutations or other modifications into the host organism by accident.

One of these strategies is base editing, which uses a deaminase enzyme to instantly convert a particular target nucleotide to its complementary base without the requirement for DSBs. The medical field has many possible uses for base editing, including therapy for treating superbug infections. Base editing is replacing conventional CRISPR-Cas methods, which involve inserting foreign DNA into the bacterial genome. By turning off the resistance genes in bacteria, base editing can restore the efficacy of existing antibiotics and thus be utilized to combat superbugs.

Additionally, BEs permit high-throughput, genome-wide genetic modification in microorganisms. *E. coli*, *B. melitensis*, *S. aureus*, *C. beijerinckii*, *K. pneumonia*, and *P. aeruginosa* are just a few of the MDR bacteria that have exhibited genome-editing success using the BE3 method. Editing effectiveness varied from 20% to 100% across these species [66,71,89,90,91].

CRISPR base editing has shown promise as a method for combating drug-resistant bacteria, and it also has the potential to be utilized to engineer bacteria with enhanced capabilities. It could be used to improve bacteria’s production of biofuels or other useful compounds [130,131]. To improve crop production and safeguard against certain diseases, CRISPR base editing could become an essential tool by allowing the creation of bacteria that are resistant to particular diseases or environmental stressors. Besides the above advantages, there are additional crucial concerns that need to be addressed in the future, i.e., ethical concerns and regulated use of CRSIPR-Cas. Genetic use restriction technology (GURT) and anti-CRISPR proteins are crucial ethical components in the application of CRISPR-Cas and gene-editing technologies [132,133,134]. GURT, often referred to as “terminator technology”, addresses concerns about the unintended spread of genetically modified organisms by rendering their seeds or offspring sterile. This technology acts as a safeguard against potential ecological disruptions. On the other hand, anti-CRISPR proteins, whether naturally occurring or engineered, serve as ethical tools in gene editing. They offer oversight, allowing researchers to reversibly control CRISPR-Cas systems, enhancing safety and precision. Anti-CRISPR proteins also stimulate discussions about the dual-use nature of CRISPR technology, prompting considerations of responsible regulation to prevent misuse while supporting their responsible use for safety and ethical monitoring in research and therapies. These ethical considerations play a vital role in the responsible and balanced advancement of gene-editing technologies.

## 7. Conclusions

The global threat posed by multidrug-resistant bacterial strains, or superbugs, is an urgent and escalating challenge exacerbated by migration and urbanization. The CRISPR-Cas system, particularly type II, is a promising frontier in the battle against antibiotic-resistant bacteria. However, realizing its full potential requires concerted efforts to address its limitations, notably toxicity and inefficiency in gene-editing applications. Enter base-editing techniques, exemplified by adenine base editors (ABEs) and cytidine base editors (CBEs), which offer precision and efficacy by directly altering single base pairs without inducing double-strand breaks (DSBs). These advancements hold the key to reshaping the landscape of antibiotic resistance. By harnessing the power of CBEs and ABEs, we can rewrite the genetic code of drug-resistant microbes, rendering them susceptible to conventional antibiotics once more. This review has illuminated the hurdles the CRISPR-Cas system faces and unveiled promising strategies to overcome them through diverse base-editing techniques. Moreover, we have explored recent breakthroughs demonstrating the capability of base editors to eliminate drug-resistant pathogens, providing a glimmer of hope in the battle against superbugs. As we venture forward, we must continue refining these cutting-edge tools to safeguard global health and combat the menace of multidrug resistance, while considering the limits of ethical concerns and the use of GURT and anti-CRISPR to limit misuse.

## Figures and Tables

**Table 1:** Features of the Cas9, Cas12, and Cas13 orthologs that are employed to modify the genomes of superbugs.

Cas Nuclease Orthologs	Origin	Function
SpCas9	*Streptococcus pyogenes*	Mediated double-stranded DNA break (DSB) creation with blunt end formation.
SpCas9-VQR		It can successfully target and cleave DNA at sites containing up to three mismatches.
dCas9		Control on or off gene expression.
xCas9		It can recognize PAM sequences that are up to five base pairs in length, compared with the three-base-pair PAM sequences required by traditional Cas9.
S_P_G		It only needs a G nucleotide to make edits.
S_P_RY		It can take advantage of various protospacer adjacent motif site sequences to modify the genetic material of bacteria.
Cas9n		Nickase activity is used to create a single-stranded break in the target DNA by cutting specific parts of the DNA sequence.
St1Cas9	*Streptococcus thermophilus*	This allows for better targeting of DNA, reducing unintended effects.
SaCas9	*Staphylococcus aureus*	Similar to SpCas9, but smaller and easier to deliver via viral or other vector-based systems.
NmCas9	*Neisseria meningitidis*	It improves specificity and reduces off-target effects.
CjCas9	*Campylobacter jejuni*	It has lower cleavage activity than spCas9, making it more suitable for applications that require fewer DNA edits.
Cpf1-Cas12a	*Prevoltella* and *Francisella*	It has a protospacer adjacent motif (PAM) recognition pattern that allows it to cut DNA at multiple sites in the target sequence.
C2c2-Cas12b	*Aquifex aeolicus*	Cuts DNA at a specific point and requires a guide RNA to direct it to the appropriate target.
AsCpf1-Cas12c	*Acidaminococcus* sp.	Smaller editing window, making it ideal for very precise gene-editing applications.
Cas13a	*Leptotrichia wadei*	RNA editing can be used for gene silencing through RNA interference.

**Table 2:** Characteristics of DNA base editors used for superbug genome editing.

DNA Base Editors	Cas Nuclease	PAM	Editing Window	Delivery Method	Use for Superbugs	Refs.
Cytosine base editors						
BE1	SpCas9	NGG	4–8	Plasmid	Yes	[65]
BE2	SpCas9	NGG	4–8	Plasmid	Yes	[66]
BE3	SpCas9	NGG	4–8	AVV, Plasmid, RNP	Yes	[66]
BE3	SaCas9	NNGRRT	3–12	Electroporation	Yes	[67]
KKH-BE3	SpCas9	NNNRRT	3–12	AAV	Yes	[67]
ea3a-BE3	SpCas9	NGG	4–8	Lentivirus	Not listed	[68]
BE3-VQR	SpCas9	NGAN	4–11	mRNA	Not listed	[68]
BE3-VRER	SpCas9	NGCG	3–10	AAV	Not listed	[68]
BE3-YEE	SpCas9	NGG	5–6	AAV	Yes	[69,70]
BE3-YE1	SpCas9	NGG	4–7	Plasmid	Yes	[69,70]
BE3-YE2	SpCas9		5–6		Yes	[69,70]
BE4	SpCas9	NGG	4–8	Plasmid, RNP	Yes	[71,72,73,74,75]
BE4	SaCas9		3–12	Plasmid	Yes	[71,72,73,74,75]
BE4-max	SauriCas9	NNGG	6–9	AAV	Not listed	[76]
Target-AID	SpCas9-NG	NG	2–4	Lentivirus	Yes	[77]
Target-AID	SpCas9	NGG	2–4	Lentivirus	Yes	[78]
Target-AID	dCas9	NGG	13–17	Plasmid	Yes	[79]
BE-Cpf1	Cas12a	TTTV	8–13	Plasmid	Yes	[80,81,82]
Adenine base editors						
ABE7.8	-	CCG	4–9	Plasmid	Yes	[83,84]
ABE7.9	Cas9	NGG	4–9	Plasmid	Yes	[83,84]
ABE7.10	ScCas9	NGG	4–8	AVV	Yes	[83,84]
ABE7.10	SpCas9D10A	NGA	4–8	Plasmid	Yes	[77]
ABE8e	SaCas9/Cas12a	-	4–8	RNP	Yes	[85]

## References

[1] Tacconelli E., Carrara E., Savoldi A., Harbarth S., Mendelson M., Monnet D.L., Pulcini C., Kahlmeter G., Kluytmans J., Carmeli Y. (2018). Discovery, research, and development of new antibiotics: The WHO priority list of antibiotic-resistant bacteria and tuberculosis. Lancet Infect. Dis..

[2] Reygaert W.C. (2018). An overview of the antimicrobial resistance mechanisms of bacteria. AIMS Microbiol..

[3] Davies J., Davies D. (2010). Origins and evolution of antibiotic resistance. Microbiol. Mol. Biol. Rev..

[4] Sun D., Jeannot K., Xiao Y., Knapp C.W. (2019). Editorial: Horizontal gene transfer mediated bacterial antibiotic resistance. Front. Microbiol..

[5] Murray J.L., Kwon T., Marcotte E.M., Whiteley M. (2015). Intrinsic antimicrobial resistance determinants in the superbug *Pseudomonas aeruginosa*. MBio.

[6] Lewis K. (2007). Persister cells, dormancy and infectious disease. Nat. Rev. Microbiol..

[7] Annunziato G. (2019). Strategies to overcome antimicrobial resistance (AMR) making use of non-essential target inhibitors: A review. Int. J. Mol. Sci..

[8] Fischbach M.A. (2011). Combination therapies for combating antimicrobial resistance. Curr. Opin. Microbiol..

[9] Buckner M.M.C., Ciusa M.L., Piddock L.J.V. (2018). Strategies to combat antimicrobial resistance: Anti-plasmid and plasmid curing. FEMS Microbiol. Rev..

[10] Gray D.A., Wenzel M. (2020). Multitarget approaches against multiresistant superbugs. ACS Infect. Dis..

[11] Ghosh C., Sarkar P., Issa R., Haldar J. (2019). Alternatives to conventional antibiotics in the era of antimicrobial resistance. Trends Microbiol..

[12] Mulani M.S., Kamble E.E., Kumkar S.N., Tawre M.S., Pardesi K.R. (2019). Emerging strategies to combat ESKAPE pathogens in the era of antimicrobial resistance: A review. Front. Microbiol..

[13] Alaoui Mdarhri H., Benmessaoud R., Yacoubi H., Seffar L., Guennouni Assimi H., Hamam M., Boussettine R., Filali-Ansari N., Lahlou F.A., Diawara I. (2022). Alternatives Therapeutic Approaches to Conventional Antibiotics: Advantages, Limitations and Potential Application in Medicine. Antibiotics.

[14] Suh G.A., Lodise T.P., Tamma P.D., Knisely J.M., Alexander J., Aslam S., Barton K.D., Bizzell E., Totten K.M.C., Campbell J.L. (2022). Considerations for the use of phage therapy in clinical practice. Antimicrob. Agents Chemother..

[15] Tetsch L. (2017). The adaptive bacterial immune system CRISPR-Cas and its therapeutic potential. Med. Monatsschr. Pharm..

[16] Wu Y., Battalapalli D., Hakeem M.J., Selamneni V., Zhang P., Draz M.S., Ruan Z. (2021). Engineered CRISPR-Cas systems for the detection and control of antibiotic-resistant infections. J. Nanobiotechnol..

[17] Sun L., He T., Zhang L., Pang M., Zhang Q., Zhou Y., Bao H., Wang R. (2017). Generation of newly discovered resistance gene mcr-1 knockout in *Escherichia coli* using the CRISPR/Cas9 system. J. Microbiol. Biotechnol..

[18] Wei W., Zhang S., Fleming J., Chen Y., Li Z., Fan S., Liu Y., Wang W., Wang T., Liu Y. (2019). *Mycobacterium tuberculosis* type III-A CRISPR/Cas system crRNA and its maturation have atypical features. FASEB J..

[19] Liu T., Pan S., Li Y., Peng N., She Q. (2018). Type III CRISPR/Cas system: Introduction and its application for genetic manipulations. Curr. Issues Mol. Biol..

[20] Lier C., Baticle E., Horvath P., Haguenoer E., Valentin A.-S., Glaser P., Mereghetti L., Lanotte P. (2015). Analysis of the type II-A CRISPR-Cas system of *Streptococcus agalactiae* reveals distinctive features according to genetic lineages. Front. Genet..

[21] Chen H., Li B., Shi S., Zhou T., Wang X., Wang Z., Zhou X., Wang M., Shi W., Ren L. (2023). Au–Fe_3_O_4_ nanozyme coupled with CRISPR-Cas12a for sensitive and visual antibiotic resistance diagnosing. Anal. Chim. Acta.

[22] Javaid N., Choi S. (2021). CRISPR/Cas system and factors affecting its precision and efficiency. Front. Cell Dev. Biol..

[23] Kundar R., Gokarn K. (2022). CRISPR-Cas System: A Tool to Eliminate Drug-Resistant Gram-Negative Bacteria. Pharmaceuticals.

[24] Kantor A., McClements M.E., MacLaren R.E. (2020). CRISPR-Cas9 DNA base-editing and prime-editing. Int. J. Mol. Sci..

[25] Ishino Y., Krupovic M., Forterre P. (2018). History of CRISPR-Cas from encounter with a mysterious repeated sequence to genome editing technology. J. Bacteriol..

[26] Wu S.-S., Li Q.-C., Yin C.-Q., Xue W., Song C.-Q. (2020). Advances in CRISPR/Cas-based gene therapy in human genetic diseases. Theranostics.

[27] Arora L., Narula A. (2017). Gene editing and crop improvement using CRISPR-Cas9 system. Front. Plant Sci..

[28] Yao R., Liu D., Jia X., Zheng Y., Liu W., Xiao Y. (2018). CRISPR-Cas9/Cas12a biotechnology and application in bacteria. Synth. Syst. Biotechnol..

[29] Tenover F.C. (2006). Mechanisms of antimicrobial resistance in bacteria. Am. J. Med..

[30] Haider M.Z., Shabbir M.A.B., Yaqub T., Sattar A., Maan M.K., Mahmood S., Mehmood T., Aslam H. (2022). Bin CRISPR-Cas system: An adaptive immune system’s association with antibiotic resistance in *Salmonella enterica* serovar enteritidis. BioMed Res. Int..

[31] van Belkum A., Soriaga L.B., LaFave M.C., Akella S., Veyrieras J.-B., Barbu E.M., Shortridge D., Blanc B., Hannum G., Zambardi G. (2015). Phylogenetic distribution of CRISPR-Cas systems in antibiotic-resistant *Pseudomonas aeruginosa*. MBio.

[32] Bikard D., Euler C.W., Jiang W., Nussenzweig P.M., Goldberg G.W., Duportet X., Fischetti V.A., Marraffini L.A. (2014). Exploiting CRISPR-Cas nucleases to produce sequence-specific antimicrobials. Nat. Biotechnol..

[33] Wang X., Lyu Y., Wang S., Zheng Q., Feng E., Zhu L., Pan C., Wang S., Wang D., Liu X. (2021). Application of CRISPR/Cas9 system for Plasmid elimination and bacterial killing of *Bacillus cereus* group strains. Front. Microbiol..

[34] Jiang W., Bikard D., Cox D., Zhang F., Marraffini L.A. (2013). RNA-guided editing of bacterial genomes using CRISPR-Cas systems. Nat. Biotechnol..

[35] Aparicio T., de Lorenzo V., Martínez-García E. (2018). CRISPR/Cas9-based counterselection boosts recombineering efficiency in *Pseudomonas putida*. Biotechnol. J..

[36] Bikard D., Hatoum-Aslan A., Mucida D., Marraffini L.A. (2012). CRISPR interference can prevent natural transformation and virulence acquisition during in vivo bacterial infection. Cell Host Microbe.

[37] de Maat V., Stege P.B., Dedden M., Hamer M., van Pijkeren J.-P., Willems R.J.L., van Schaik W. (2019). CRISPR-Cas9-mediated genome editing in vancomycin-resistant *Enterococcus faecium*. FEMS Microbiol. Lett..

[38] Zetsche B., Heidenreich M., Mohanraju P., Fedorova I., Kneppers J., DeGennaro E.M., Winblad N., Choudhury S.R., Abudayyeh O.O., Gootenberg J.S. (2017). Erratum: Multiplex gene editing by CRISPR–Cpf1 using a single crRNA array. Nat. Biotechnol..

[39] Hillary V.E., Ceasar S.A. (2023). A Review on the Mechanism and Applications of CRISPR/Cas9/Cas12/Cas13/Cas14 Proteins Utilized for Genome Engineering. Mol. Biotechnol..

[40] Li Y., Shi Z., Hu A., Cui J., Yang K., Liu Y., Deng G., Zhu C., Zhu L. (2022). Rapid One-Tube RPA-CRISPR/Cas12 Detection Platform for Methicillin-Resistant *Staphylococcus aureus*. Diagnostics.

[41] Wang Y., Guo Y., Zhang L., Yang Y., Yang S., Yang L., Chen H., Liu C., Li J., Xie G. (2021). Integration of multiplex PCR and CRISPR-Cas allows highly specific detection of multidrug-resistant Acinetobacter Baumannii. Sens. Actuators B Chem..

[42] Jiang Y., Qian F., Yang J., Liu Y., Dong F., Xu C., Sun B., Chen B., Xu X., Li Y. (2017). CRISPR-Cpf1 assisted genome editing of *Corynebacterium glutamicum*. Nat. Commun..

[43] Wang S., Wang S., Tang Y., Peng G., Hao T., Wu X., Wei J., Qiu X., Zhou D., Zhu S. (2023). Detection of *Klebsiella pneumonia* DNA and ESBL positive strains by PCR-based CRISPR-LbCas12a system. Front. Microbiol..

[44] Mukama O., Wu J., Li Z., Liang Q., Yi Z., Lu X., Liu Y., Liu Y., Hussain M., Makafe G.G. (2020). An ultrasensitive and specific point-of-care CRISPR/Cas12 based lateral flow biosensor for the rapid detection of nucleic acids. Biosens. Bioelectron..

[45] Kordyś M., Sen R., Warkocki Z. (2022). Applications of the versatile CRISPR-Cas13 RNA targeting system. Wiley Interdiscip. Rev. RNA.

[46] Kiga K., Tan X.-E., Ibarra-Chávez R., Watanabe S., Aiba Y., Sato’o Y., Li F.-Y., Sasahara T., Cui B., Kawauchi M. (2020). Development of CRISPR-Cas13a-based antimicrobials capable of sequence-specific killing of target bacteria. Nat. Commun..

[47] Palacios Araya D., Palmer K.L., Duerkop B.A. (2021). CRISPR-based antimicrobials to obstruct antibiotic-resistant and pathogenic bacteria. PLoS Pathog..

[48] Jinek M., Chylinski K., Fonfara I., Hauer M., Doudna J.A., Charpentier E. (2012). A programmable dual-RNA–guided DNA endonuclease in adaptive bacterial immunity. Science.

[49] Kohm K., Basu S., Nawaz M.M., Hertel R. (2021). Chances and limitations when uncovering essential and non-essential genes of *Bacillus subtilis* phages with CRISPR-Cas9. Environ. Microbiol. Rep..

[50] Schilling T., Hoppert M., Hertel R. (2018). Genomic analysis of the recent viral isolate vB_BthP-Goe4 reveals increased diversity of φ29-like phages. Viruses.

[51] Chatterjee P., Jakimo N., Jacobson J.M. (2018). Minimal PAM specificity of a highly similar SpCas9 ortholog. Sci. Adv..

[52] Sheel A., Xue W. (2016). Genomic amplifications cause false positives in CRISPR screens. Cancer Discov..

[53] Zhang Y., Qin W., Lu X., Xu J., Huang H., Bai H., Li S., Lin S. (2017). Programmable base editing of zebrafish genome using a modified CRISPR-Cas9 system. Nat. Commun..

[54] Mishra R., Joshi R.K., Zhao K. (2020). Base editing in crops: Current advances, limitations and future implications. Plant Biotechnol. J..

[55] Lim C.K.W., Gapinske M., Brooks A.K., Woods W.S., Powell J.E., Winter J., Perez-Pinera P., Gaj T. (2020). Treatment of a mouse model of ALS by in vivo base editing. Mol. Ther..

[56] Pulecio J., Verma N., Mejía-Ramírez E., Huangfu D., Raya A. (2017). CRISPR/Cas9-based engineering of the epigenome. Cell Stem Cell.

[57] Gasiunas G., Barrangou R., Horvath P., Siksnys V. (2012). Cas9–crRNA ribonucleoprotein complex mediates specific DNA cleavage for adaptive immunity in bacteria. Proc. Natl. Acad. Sci. USA.

[58] Liu X., Zhang Y., Chen Y., Li M., Zhou F., Li K., Cao H., Ni M., Liu Y., Gu Z. (2017). In situ capture of chromatin interactions by biotinylated dCas9. Cell.

[59] Kuncheva E. (2022). Activation of KCNQ1 Expression in HEK293 Cells Using Inducible CRISPR-dCas9-VPR Tripartite Transcriptional Activator Domain. Master’s Thesis.

[60] Das S., Bano S., Kapse P., Kundu G.C. (2022). CRISPR based therapeutics: A new paradigm in cancer precision medicine. Mol. Cancer.

[61] Lan T.-H., He L., Huang Y., Zhou Y. (2022). Optogenetics for transcriptional programming and genetic engineering. Trends Genet..

[62] Lainšček D., Kadunc L., Keber M.M., Bratkovič I.H., Romih R., Jerala R. (2018). Delivery of an artificial transcription regulator dCas9-VPR by extracellular vesicles for therapeutic gene activation. ACS Synth. Biol..

[63] Li A., Cartwright S., Yu A., Ho S.-M., Schrode N., Deans P.J.M., Matos M.R., Garcia M.F., Townsley K.G., Zhang B. (2021). Using the dCas9-KRAB system to repress gene expression in hiPSC-derived NGN2 neurons. STAR Protoc..

[64] Tadić V., Josipović G., Zoldoš V., Vojta A. (2019). CRISPR/Cas9-based epigenome editing: An overview of dCas9-based tools with special emphasis on off-target activity. Methods.

[65] Zhong Z., Guo J., Deng L., Chen L., Wang J., Li S., Xu W., Deng Z., Sun Y. (2019). Base editing in *Streptomyces* with Cas9-deaminase fusions. BioRxiv.

[66] Zheng K., Wang Y., Li N., Jiang F.-F., Wu C.-X., Liu F., Chen H.-C., Liu Z.-F. (2018). Highly efficient base editing in bacteria using a Cas9-cytidine deaminase fusion. Commun. Biol..

[67] Kleinstiver B.P., Prew M.S., Tsai S.Q., Nguyen N.T., Topkar V.V., Zheng Z., Joung J.K. (2015). Broadening the targeting range of *Staphylococcus aureus* CRISPR-Cas9 by modifying PAM recognition. Nat. Biotechnol..

[68] Kim Y.B., Komor A.C., Levy J.M., Packer M.S., Zhao K.T., Liu D.R. (2017). Increasing the genome-targeting scope and precision of base editing with engineered Cas9-cytidine deaminase fusions. Nat. Biotechnol..

[69] Jiang L., Long J., Yang Y., Zhou L., Su J., Qin F., Tang W., Tao R., Chen Q., Yao S. (2022). Internally inlaid SaCas9 base editors enable window specific base editing. Theranostics.

[70] Doman J.L., Raguram A., Newby G.A., Liu D.R. (2020). Evaluation and minimization of Cas9-independent off-target DNA editing by cytosine base editors. Nat. Biotechnol..

[71] Li Q., Seys F.M., Minton N.P., Yang J., Jiang Y., Jiang W., Yang S. (2019). CRISPR–Cas9D10A nickase-assisted base editing in the solvent producer *Clostridium beijerinckii*. Biotechnol. Bioeng..

[72] Sun J., Lu L.-B., Liang T.-X., Yang L.-R., Wu J.-P. (2020). CRISPR-assisted multiplex base editing system in *Pseudomonas putida* KT2440. Front. Bioeng. Biotechnol..

[73] Yue S., Huang P., Li S., Cai Y., Wang W., Zhang X., Nikel P.I., Hu H. (2022). Developing a CRISPR-assisted base-editing system for genome engineering of *Pseudomonas chlororaphis*. Microb. Biotechnol..

[74] Lam D.K., Feliciano P.R., Arif A., Bohnuud T., Fernandez T.P., Gehrke J.M., Grayson P., Lee K.D., Ortega M.A., Sawyer C. (2023). Improved cytosine base editors generated from TadA variants. Nat. Biotechnol..

[75] Shelake R.M., Pramanik D., Kim J.-Y. (2023). Improved Dual Base Editor Systems (iACBEs) for Simultaneous Conversion of Adenine and Cytosine in the Bacterium *Escherichia coli*. MBio.

[76] Hu Z., Wang S., Zhang C., Gao N., Li M., Wang D., Wang D., Liu D., Liu H., Ong S.-G. (2020). A compact Cas9 ortholog from *Staphylococcus Auricularis* (SauriCas9) expands the DNA targeting scope. PLoS Biol..

[77] Zhang Y., Zhang H., Wang Z., Wu Z., Wang Y., Tang N., Xu X., Zhao S., Chen W., Ji Q. (2020). Programmable adenine deamination in bacteria using a Cas9–adenine-deaminase fusion. Chem. Sci..

[78] Banno S., Nishida K., Arazoe T., Mitsunobu H., Kondo A. (2018). Deaminase-mediated multiplex genome editing in *Escherichia coli*. Nat. Microbiol..

[79] Zhao Y., Tian J., Zheng G., Chen J., Sun C., Yang Z., Zimin A.A., Jiang W., Deng Z., Wang Z. (2020). Multiplex genome editing using a dCas9-cytidine deaminase fusion in *Streptomyces*. Sci. China Life Sci..

[80] Li X., Wang Y., Liu Y., Yang B., Wang X., Wei J., Lu Z., Zhang Y., Wu J., Huang X. (2018). Base editing with a Cpf1–cytidine deaminase fusion. Nat. Biotechnol..

[81] Zhang J., Hong W., Zong W., Wang P., Wang Y. (2018). Markerless genome editing in *Clostridium beijerinckii* using the CRISPR-Cpf1 system. J. Biotechnol..

[82] Hong W., Zhang J., Cui G., Wang L., Wang Y. (2018). Multiplexed CRISPR-Cpf1-mediated genome editing in *Clostridium difficile* toward the understanding of pathogenesis of *C. difficile* infection. ACS Synth. Biol..

[83] Wu W., Yang Y., Lei H. (2019). Progress in the application of CRISPR: From gene to base editing. Med. Res. Rev..

[84] Zhao X., Sun Z., Kang W., Tao Y., Wu H. (2020). A review of application of base editing for the treatment of inner ear disorders. J. Bio-X Res..

[85] Richter M.F., Zhao K.T., Eton E., Lapinaite A., Newby G.A., Thuronyi B.W., Wilson C., Koblan L.W., Zeng J., Bauer D.E. (2020). Phage-assisted evolution of an adenine base editor with improved Cas domain compatibility and activity. Nat. Biotechnol..

[86] Komor A.C., Badran A.H., Liu D.R. (2017). CRISPR-based technologies for the manipulation of eukaryotic genomes. Cell.

[87] Chiu Y.-L., Greene W.C. (2006). Multifaceted antiviral actions of APOBEC3 cytidine deaminases. Trends Immunol..

[88] Wang Y., Wang S., Chen W., Song L., Zhang Y., Shen Z., Yu F., Li M., Ji Q. (2018). Precise and efficient genome editing in *Klebsiella pneumoniae* using CRISPR-Cas9 and CRISPR-assisted cytidine deaminase. Appl. Environ. Microbiol..

[89] Sun Q., Wang Y., Dong N., Shen L., Zhou H., Hu Y., Gu D., Chen S., Zhang R., Ji Q. (2019). Application of CRISPR/Cas9-based genome editing in studying the mechanism of pandrug resistance in Klebsiella pneumoniae. Antimicrob. Agents Chemother..

[90] Chen W., Zhang Y., Zhang Y., Pi Y., Gu T., Song L., Wang Y., Ji Q. (2018). CRISPR/Cas9-based genome editing in *Pseudomonas aeruginosa* and cytidine deaminase-mediated base editing in *Pseudomonas* species. IScience.

[91] Gu T., Zhao S., Pi Y., Chen W., Chen C., Liu Q., Li M., Han D., Ji Q. (2018). Highly efficient base editing in *Staphylococcus aureus* using an engineered CRISPR RNA-guided cytidine deaminase. Chem. Sci..

[92] Wang Y., Wang Z., Chen Y., Hua X., Yu Y., Ji Q. (2019). A highly efficient CRISPR-Cas9-based genome engineering platform in *Acinetobacter baumannii* to understand the H_2_O_2_-sensing mechanism of OxyR. Cell Chem. Biol..

[93] Tong Y., Whitford C.M., Robertsen H.L., Blin K., Jørgensen T.S., Klitgaard A.K., Gren T., Jiang X., Weber T., Lee S.Y. (2019). Highly efficient DSB-free base editing for streptomycetes with CRISPR-BEST. Proc. Natl. Acad. Sci. USA.

[94] Ding X.-Y., Li S.-S., Geng Y.-M., Yan M.-Y., Li G.-B., Zhang G.-L., Sun Y.-C. (2021). Programmable base editing in *Mycobacterium tuberculosis* using an engineered CRISPR RNA-guided cytidine deaminase. Front. Genome Ed..

[95] Zhang H., Zhang Y., Wang W.-X., Chen W., Zhang X., Huang X., Chen W., Ji Q. (2022). Pam-expanded streptococcus thermophilus cas9 c-to-t and c-to-g base editors for programmable base editing in mycobacteria. Engineering.

[96] Gaudelli N.M., Komor A.C., Rees H.A., Packer M.S., Badran A.H., Bryson D.I., Liu D.R. (2017). Programmable base editing of A• T to G• C in genomic DNA without DNA cleavage. Nature.

[97] Kim J., Malashkevich V., Roday S., Lisbin M., Schramm V.L., Almo S.C. (2006). Structural and kinetic characterization of *Escherichia coli* TadA, the wobble-specific tRNA deaminase. Biochemistry.

[98] Wolf J., Gerber A.P., Keller W. (2002). tadA, an essential tRNA-specific adenosine deaminase from *Escherichia coli*. EMBO J..

[99] Abdullah, Wang P., Han T., Liu W., Ren W., Wu Y., Xiao Y. (2022). Adenine base editing system for *Pseudomonas* and prediction workflow for protein dysfunction via ABE. ACS Synth. Biol..

[100] Grünewald J., Zhou R., Lareau C.A., Garcia S.P., Iyer S., Miller B.R., Langner L.M., Hsu J.Y., Aryee M.J., Joung J.K. (2020). A dual-deaminase CRISPR base editor enables concurrent adenine and cytosine editing. Nat. Biotechnol..

[101] Sakata R.C., Ishiguro S., Mori H., Tanaka M., Tatsuno K., Ueda H., Yamamoto S., Seki M., Masuyama N., Nishida K. (2020). Base editors for simultaneous introduction of C-to-T and A-to-G mutations. Nat. Biotechnol..

[102] Xu R., Kong F., Qin R., Li J., Liu X., Wei P. (2021). Development of an efficient plant dual cytosine and adenine editor. J. Integr. Plant Biol..

[103] Hao W., Cui W., Suo F., Han L., Cheng Z., Zhou Z. (2022). Construction and application of an efficient dual-base editing platform for *Bacillus subtilis* evolution employing programmable base conversion. Chem. Sci..

[104] Lim C.K.W., Miskalis A.J., Perez-Pinera P., Gaj T. (2023). Delivering Base Editors In Vivo by Adeno-Associated Virus Vectors. Base Editors: Methods and Protocols.

[105] Li C., Samulski R.J. (2020). Engineering adeno-associated virus vectors for gene therapy. Nat. Rev. Genet..

[106] Sargiannidou I., Kagiava A., Kleopa K.A. (2020). Gene therapy approaches targeting Schwann cells for demyelinating neuropathies. Brain Res..

[107] Asokan A., Schaffer D.V., Samulski R.J. (2012). The AAV vector toolkit: Poised at the clinical crossroads. Mol. Ther..

[108] Kweon J., Jang A.-H., Kwon E., Kim U., Shin H.R., See J., Jang G., Lee C., Koo T., Kim S. (2023). Targeted dual base editing with *Campylobacter jejuni* Cas9 by single AAV-mediated delivery. Exp. Mol. Med..

[109] Zetsche B., Volz S.E., Zhang F. (2015). A split-Cas9 architecture for inducible genome editing and transcription modulation. Nat. Biotechnol..

[110] Li Y. (2015). Split-inteins and their bioapplications. Biotechnol. Lett..

[111] Zhi S., Chen Y., Wu G., Wen J., Wu J., Liu Q., Li Y., Kang R., Hu S., Wang J. (2022). Dual-AAV delivering split prime editor system for in vivo genome editing. Mol. Ther..

[112] Volkmann G., Iwai H. (2010). Protein trans-splicing and its use in structural biology: Opportunities and limitations. Mol. Biosyst..

[113] López-Igual R., Bernal-Bayard J., Rodríguez-Patón A., Ghigo J.-M., Mazel D. (2019). Engineered toxin–intein antimicrobials can selectively target and kill antibiotic-resistant bacteria in mixed populations. Nat. Biotechnol..

[114] Khoshandam M., Soltaninejad H., Mousazadeh M., Hamidieh A.A., Hosseinkhani S. (2023). Clinical applications of the CRISPR/Cas9 genome-editing system: Delivery options and challenges in precision medicine. Genes Dis..

[115] Pensado A., Seijo B., Sanchez A. (2014). Current strategies for DNA therapy based on lipid nanocarriers. Expert Opin. Drug Deliv..

[116] Kang Y.K., Kwon K., Ryu J.S., Lee H.N., Park C., Chung H.J. (2017). Nonviral genome editing based on a polymer-derivatized CRISPR nanocomplex for targeting bacterial pathogens and antibiotic resistance. Bioconjug. Chem..

[117] Lino C.A., Harper J.C., Carney J.P., Timlin J.A. (2018). Delivering CRISPR: A review of the challenges and approaches. Drug Deliv..

[118] Kolasinliler G., Aagre M.M., Akkale C., Kaya H.B. (2023). The use of CRISPR-Cas-based systems in bacterial cell factories. Biochem. Eng. J..

[119] Guzmán-Zapata D., Sandoval-Vargas J.M., Macedo-Osorio K.S., Salgado-Manjarrez E., Castrejón-Flores J.L., Oliver-Salvador M.d.C., Durán-Figueroa N.V., Nogué F., Badillo-Corona J.A. (2019). Efficient editing of the nuclear APT reporter gene in *Chlamydomonas reinhardtii* via expression of a CRISPR-Cas9 module. Int. J. Mol. Sci..

[120] Khodakova A.S., Vilchis D.V., Blackburn D., Amanor F., Samuel B.S. (2021). Population scale nucleic acid delivery to *Caenorhabditis elegans* via electroporation. G3.

[121] Singh I., Arora R., Dhiman H., Pahwa R. (2018). Carbon quantum dots: Synthesis, characterization and biomedical applications. Turk. J. Pharm. Sci.

[122] Kalashgrani M.Y., Nejad F.F., Rahmanian V. (2022). Carbon Quantum Dots Platforms: As nano therapeutic for Biomedical Applications. Adv. Appl. NanoBio-Technol..

[123] Mazumdar A., Haddad Y., Milosavljevic V., Michalkova H., Guran R., Bhowmick S., Moulick A. (2020). Peptide-carbon quantum dots conjugate, derived from human retinoic acid receptor responder protein 2, against antibiotic-resistant gram positive and gram negative pathogenic bacteria. Nanomaterials.

[124] Zhao C., Wu L., Wang X., Weng S., Ruan Z., Liu Q., Lin L., Lin X. (2020). Quaternary ammonium carbon quantum dots as an antimicrobial agent against gram-positive bacteria for the treatment of MRSA-infected pneumonia in mice. Carbon.

[125] Huang S., Zhang Q., Liu P., Ma S., Xie B., Yang K., Zhao Y. (2020). Novel up-conversion carbon quantum dots/α-FeOOH nanohybrids eliminate tetracycline and its related drug resistance in visible-light responsive Fenton system. Appl. Catal. B Environ..

[126] Li P., Liu S., Cao W., Zhang G., Yang X., Gong X., Xing X. (2020). Low-toxicity carbon quantum dots derived from gentamicin sulfate to combat antibiotic resistance and eradicate mature biofilms. Chem. Commun..

[127] Li P., Liu S., Yang X., Du S., Tang W., Cao W., Zhou J., Gong X., Xing X. (2021). Low-drug resistance carbon quantum dots decorated injectable self-healing hydrogel with potent antibiofilm property and cutaneous wound healing. Chem. Eng. J..

[128] Zhang S., Shen J., Li D., Cheng Y. (2021). Strategies in the delivery of Cas9 ribonucleoprotein for CRISPR/Cas9 genome editing. Theranostics.

[129] Qiao J., Li W., Lin S., Sun W., Ma L., Liu Y. (2019). Co-expression of Cas9 and single-guided RNAs in *Escherichia coli* streamlines production of Cas9 ribonucleoproteins. Commun. Biol..

[130] Thuronyi B.W., Koblan L.W., Levy J.M., Yeh W.-H., Zheng C., Newby G.A., Wilson C., Bhaumik M., Shubina-Oleinik O., Holt J.R. (2019). Continuous evolution of base editors with expanded target compatibility and improved activity. Nat. Biotechnol..

[131] Cho S., Shin J., Cho B.-K. (2018). Applications of CRISPR/Cas system to bacterial metabolic engineering. Int. J. Mol. Sci..

[132] Aksoy E., Yildirim K., Kavas M., Kayihan C., Yerlikaya B.A., Çalik I., Sevgen İ., Demirel U. (2022). General guidelines for CRISPR/Cas-based genome editing in plants. Mol. Biol. Rep..

[133] Davidson A.R., Lu W.T., Stanley S.Y., Wang J., Mejdani M., Trost C.N., Hicks B.T., Lee J., Sontheimer E.J. (2020). Anti-CRISPRs: Protein Inhibitors of CRISPR-Cas Systems. Annu. Rev. Biochem..

[134] Tao S., Chen H., Li N., Liang W. (2022). The application of the CRISPR-Cas system in antibiotic resistance. Infect. Drug Resist..

